# lncRNA NR2F1‐AS1 promotes breast cancer angiogenesis through activating IGF‐1/IGF‐1R/ERK pathway

**DOI:** 10.1111/jcmm.15499

**Published:** 2020-06-17

**Authors:** Qi Zhang, Tianfu Li, Zhecun Wang, Xiaying Kuang, Nan Shao, Ying Lin

**Affiliations:** ^1^ Breast Disease Center The First Affiliated Hospital Sun Yat‐Sen University Guangzhou China; ^2^ National‐Guangdong Joint Engineering Laboratory for Diagnosis and Treatment of Vascular Disease The First Affiliated Hospital Sun Yat‐Sen University Guangzhou China; ^3^ Laboratory of Surgery The First Affiliated Hospital Sun Yat‐Sen University Guangzhou China; ^4^ Division of Vascular Surgery The First Affiliated Hospital Sun Yat‐Sen University Guangzhou China

**Keywords:** angiogenesis, breast cancer, IGF‐1, IGF‐1R, lncRNA NR2F1‐AS1

## Abstract

Long non‐coding RNAs (lncRNAs) take various effects in cancer mostly through sponging with microRNAs (miRNAs). lncRNA NR2F1‐AS1 is found to promote tumour progression in hepatocellular carcinoma, endometrial cancer and thyroid cancer. However, the role of lncRNA NR2F1‐AS1 in breast cancer angiogenesis remains unknown. In this study, we found lncRNA NR2F1‐AS1 was positively related with CD31 and CD34 in breast cancer through Pearson's correlation analysis, while lncRNA NR2F1‐AS1 transfection promoted human umbilical vascular endothelial cell (HUVEC) tube formation. In breast cancer cells, lncRNA NR2F1‐AS1 enhanced the HUVEC proliferation, tube formation and migration ability through tumour‐conditioned medium (TCM). In zebrafish model, lncRNA NR2F1‐AS1 increased the breast cancer cell‐related neo‐vasculature and subsequently promoted the breast cancer cell metastasis. In mouse model, lncRNA NR2F1‐AS1 promoted the tumour vessel formation, increased the micro vessel density (MVD) and then induced the growth of primary tumour. Mechanically, lncRNA NR2F1‐AS1 increased insulin‐like growth factor‐1 (IGF‐1) expression through sponging miRNA‐338‐3p in breast cancer cells and then activated the receptor of IGF‐1 (IGF‐1R) and extracellular signal‐regulated kinase (ERK) pathway in HUVECs. These results indicated that lncRNA NR2F1‐AS1 could promote breast cancer angiogenesis through IGF‐1/IGF‐1R/ERK pathway.

## 1 INTRODUCTION

Breast cancer has been a worldwide highly malignant disease for the past decades. And angiogenesis is regarded as a vital process in breast cancer development. Due to neovascularization, tumour cells could acquire essential nutrients to grow and disseminate, which results in tumour generation, progression and metastasis.^1^, ^2^ Thus, the microvessel density (MVD) is considered as a risk factor of metastasis and predicts poor prognosis of breast cancer patients.^3^, ^4^, ^5^ And breast cancer angiogenesis could act as not only potential diagnostic target but also practical therapeutic target.^6^, ^7^ However, the specific mechanism under breast cancer angiogenesis remains unclear and further study needs to be done.

Long non‐coding RNAs (lncRNAs) are a group RNAs whose length are longer than 200 base pairs without coding protein.^8^, ^9^ These RNAs widely exist in animals and humans and exert various biological effects as antisense transcripts on transcriptional regulation, cellular function and many disease including cancer.^10^, ^11^ lncRNAs take part in numerous cancer‐related procedures such as proliferation, apoptosis, stem cell differentiation, metastasis and therapy resistance.^12^, ^13^, ^14^, ^15^, ^16^ And one of the underlying mechanisms is that lncRNAs could combine with microRNAs (miRNA) as competing endogenous RNA (ceRNA) to protect the target genes.^17^, ^18^ It was reported that lncRNA NR2F1‐AS1 regulated hepatocellular carcinoma oxaliplatin resistance,^19^ and it was also involved in the progression of endometrial cancer.^20^ Moreover, lncRNA NR2F1‐AS1 was found to promote proliferation and migration yet suppress apoptosis of thyroid cancer cells through regulating miRNA‐338‐3p.^21^ However, the role of lncRNA NR2F1‐AS1 in breast cancer especially in breast cancer angiogenesis is still unknown.

Insulin‐like growth factor‐1 (IGF‐1) is a kind of small polypeptide that could regulate mammary development.^22^ Researchers found that IGF‐1 also have various biologic effects in cancer development including modulating stem cells, genomic stability, cellular metabolism and angiogenesis.^23^ In breast cancer, IGF‐1 could induce proliferation and migration,^24^, ^25^ hence high expression of IGF‐1 was related to increased risk of breast cancer,^26^ and IGF‐1 was regarded as a promising therapy target in breast cancer.^27^ IGF‐1 could also act as one of the pro‐angiogenetic factors through inducing VEGF or NO in breast cancer microenvironment.^28^, ^29^ The receptor of IGF‐1 (IGF‐1R) with the combination of IGF‐1 could exert vital function in cancer such as regulating cancer stem cell, epithelial‐mesenchymal transition and tumour microenvironment,^30^ which further influence cancer progression, metastasis and angiogenesis.^31^ However, the specific mechanism of IGF‐1 and IGF‐1R regulating breast cancer angiogenesis requires further investigation yet.

In this study, we investigated the promotive effects of lncRNA NR2F1‐AS1 on breast cancer angiogenesis both in vitro and in vivo, declaring the potential mechanism through inducing the expression of IGF‐1 in breast cancer cells and then activating IGF‐1R/ERK pathway in endothelial cells.

## 2 MATERIALS AND METHODS

### 2.1 Cell culture

Human breast cancer cell lines MDA‐MB‐231 and MCF‐7 were obtained from American Type Culture Collection (ATCC, Manassas, VA, USA) and cultured in Dulbecco's modified Eagle's medium (DMEM; Gibco, USA) with 10% foetal bovine serum (FBS; Gibco), and 1% penicillin and streptomycin (Gibco).

Human umbilical vascular endothelial cells (HUVECs) were obtained from umbilical cord and maintained in endothelial cell medium (ECM; ScienCell, USA) with 10% FBS (ScienCell), 1% endothelial cell growth supplement (ScienCell), and 1% penicillin and streptomycin (ScienCell).

All the cells were incubated in the humidified incubator at 37°C with 5% CO_2_.

### 2.2 Cell transfection

Breast cancer cells and HUVECs were transfected with lentivirus carrying specific plasmid (GeneChem, Shanghai, China) to establish MDA‐MB‐231 knock‐down of lncRNA NR2F1‐AS1 (MDA‐MB‐231‐sh‐NR2F1), MCF‐7 overexpressing lncRNA NR2F1‐AS1 (MCF‐7‐lenti‐NR2F1), HUVEC knock‐down of lncRNA NR2F1‐AS1 (HUVEC‐sh‐NR2F1), HUVECs overexpressing lncRNA NR2F1‐AS1 (HUVEC‐lenti‐NR2F1) and their control cells. Puromycin was applied to select stable transfected cells.

### 2.3 Quantitative real‐time polymerase chain reaction (qPCR)

Total RNA was extracted from human breast cancer cell lines or HUVECs through TRIzol reagent (Invitrogen, USA) and taken into reverse transcription through PrimeScript RT reagent Kit (Takara, Japan) to produce cDNA under manufacturer's instructions. qPCR was conducted through SYBR Green (Takara) and LightCycler480 system (Roche, Switzerland).

### 2.4 Tumour‐conditioned medium (TCM)

Breast cancer cells were seeded in 6‐well plates. Then, the culture medium was removed while serum‐free DMEM was added. The supernatant was collected after 48‐hours culture, centrifuged at 152 *g* for 10 minutes and filtered with 0.22 μm membrane to get TCM. The TCM was then stored at −80°C for tumour angiogenetic assays in vitro. For the tube formation assay, TCM was concentrated 75‐fold with ultrafiltration device (Millipore, USA).

### 2.5 Tube formation assay

Pre‐cooled 96‐well plate was coated with 50 μL growth factor–reduced Matrigel (BD, Corning, USA) and incubated for 30 minutes at 37°C. Serum‐free ECM starved HUVECs were seeded at 2 × 10^4^/well on the gel in 200 μL concentrated TCM (or in 200 μL DMEM containing 2% FBS for transfected HUVECs). Capillary structure was observed continuously within 12‐hour period under microscope.

### 2.6 Wound healing assay

Human umbilical vascular endothelial cells were seeded in 6‐well plates to full confluence. Vertical scratches were drawn with pipette tip, after which the culture medium was changed for TCM. The migration was observed every 24 hours for 3 days under microscope.

### 2.7 CCK8 proliferation assay

Human umbilical vascular endothelial cells were seeded in 96‐well plates to get adherent. Then, the complete medium was removed and TCM was added. CCK8 (Dojindo Laboratories, Japan) assay was performed according to manufacturer's instructions. The absorbance at 450 nm wavelength was measured every day for 5 days with microplate reader (Sunrise, Tecan, Austria).

### 2.8 Zebrafish model

Breast cancer cells were incubated with fluorescent carbocyanine dye Dil (GeneCopoeia, USA) according to manufacturer's instructions. The labelled cells were injected in the perivitelline cavity of Tg (fli1:EGFP) zebrafish embryos at 48 hours post‐fertilization using microinject system (Eppendorf, German) (for MDA‐MB‐231, n = 13/group; for MCF‐7, n = 14/group). The zebrafish embryos were observed under fluorescence microscopy, and the metastasis of breast cancer cells was measured. Vascular structure of zebrafish was observed under confocal microscope (LSM880; Zeiss, German).

### 2.9 Western blotting

Total protein was extracted from breast cancer cells with RIPA lysis buffer (Beyotime, Shanghai, China) containing phenylmethylsulphonyl fluoride (PMSF), proteinase and phosphatase inhibitors. Equal amount of protein was separated by SDS‐polyacrylamide gel and transferred to polyvinylidene fluoride (PVDF) membranes (Millipore). Blocked with bovine serum albumin (BSA, Sigma), the membranes were incubated in specific antibodies targeting IGF‐1 (R&D system, #AF‐291‐NA, 1:10 000, USA), IGF‐1R (CST, #9750, 1:1000, USA), phospho‐IGF‐1R (Tyr1135, CST, #3918, 1:1000), ERK1/2 (CST, #4695S, 1:1000) and phospho‐ERK1/2 (Thr202/Tyr204, CST, #4370P, 1:2000). After incubated in secondary antibody, the protein expression was showed using ECL luminol reagent (Millipore) by Amersham Imager 600 system (AI600, USA).

### 2.10 Dual‐luciferase reporter assay

Wide‐type IGF‐1 (IGF‐1‐WT), mutated‐type IGF‐1 (IGF‐1‐Mut), has‐miR‐338‐3p mimics and NC mimics vectors were constructed and cotransfected in 293T cells in 96‐well plates using lipo2000 (Life, USA). At 48h after transfection, the luciferase was detected using Dual‐Glo@Luciferase Assay System E2940 (Promega, USA) according to manufacturer's protocol.

### 2.11 Mouse model

NOD/SCID mice (female, 4 weeks old, GemPharmatech, China) were divided into four groups randomly as MDA‐MB‐231‐sh‐NC, MDA‐MB‐231‐sh‐NR2F1, MCF‐7‐lenti‐Vec and MCF‐7‐lenti‐NR2F1 (n = 6/group). 5 × 10^6^ transfected cells were injected into the fat pad under the breast of each mouse. Then, mice were sacrificed and dissected at 4 weeks after injection and tumour masses were weighed and then fixed in formalin for immunohistochemical (IHC) staining. CD31 (Abcam, ab182981, 1:2000, UK) was applied for the staining of endothelial cells to evaluate the micro vessel density (MVD).

### 2.12 Bioinformatics analysis and statistical analysis

The Pearson correlation analysis was conducted by Gene Expression Profiling Interactive Analysis (GEPIA, http://gepia.cancer‐pku.cn/). The Gene Ontology (GO) enrichment and the Kyoto Encyclopedia of Genes and Genomes (KEGG) pathway analysis were performed through online software DAVID Bioinformatics Resources 6.8 (https://david.ncifcrf.gov/). The target gene was predicted by the online software TargetScan Human 7.2 (http://www.targetscan.org/vert_72/) and online software MiRanda and mirSVR (http://www.microrna.org/microrna/home.do).

All experiments were carried out for at least three times from biological level. Statistical significance was calculated by two‐tailed Student's *t* test using Prism GraphPad 7.0 software and SPSS 24.0 software. The data with *P* value <.05 were defined as statistically significant.

## 3 RESULTS

### 3.1 High lncRNA NR2F1‐AS1 expression is associated with breast cancer angiogenesis

**FIGURE 1:**
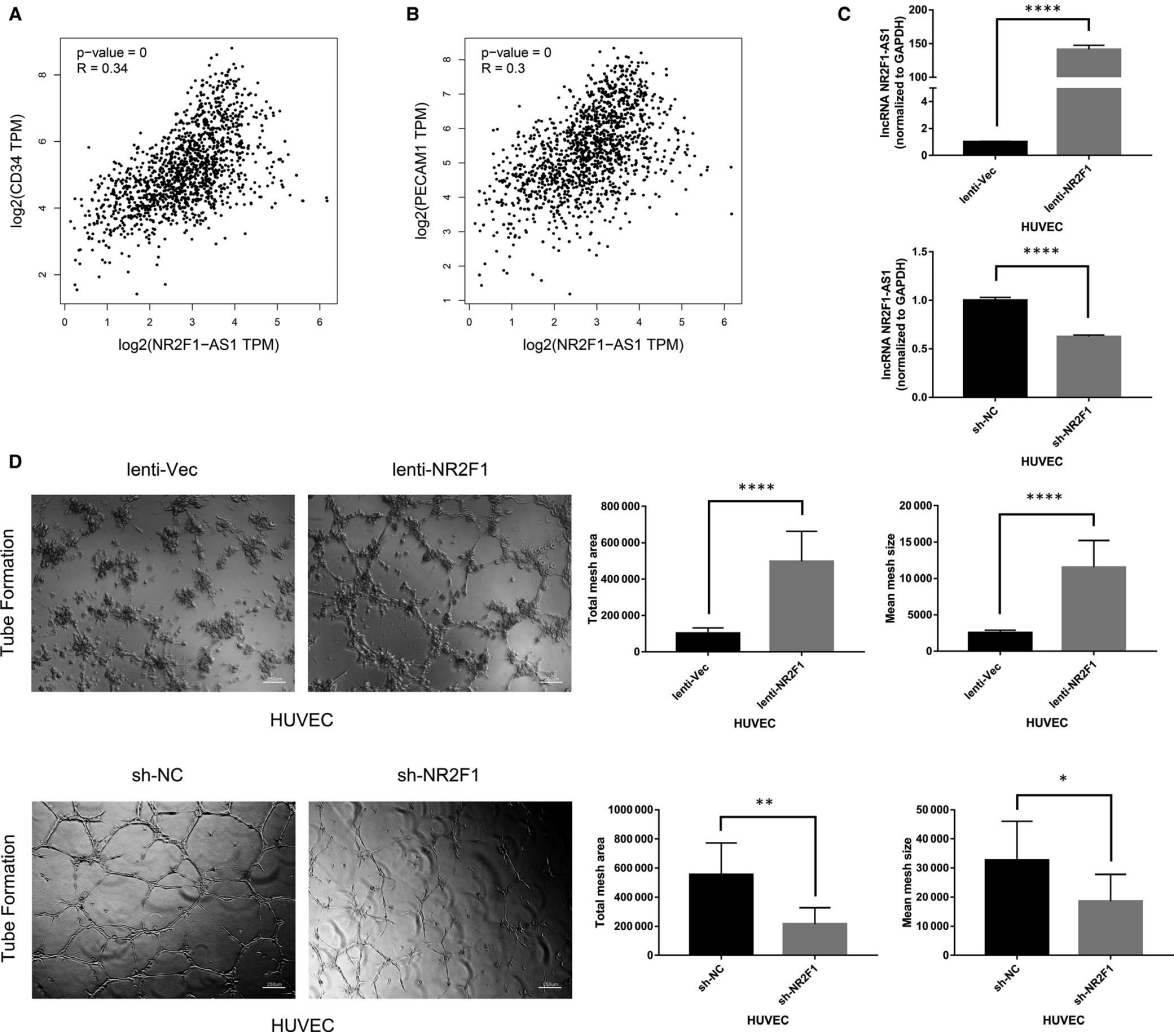
High lncRNA NR2F1‐AS1 expression is associated with breast cancer angiogenesis. A, Pearson's correlation analysis of lncRNA NR2F1‐AS1 and CD34 in breast cancer. B, Pearson's correlation analysis of lncRNA NR2F1‐AS1 and CD31 in breast cancer. C, Expression of lncRNA NR2F1‐AS1 in HUVECs overexpressing lncRNA NR2F1‐AS1 and knock‐down of lncRNA NR2F1‐AS1. D, Tube formation of transfected HUVECs (representative image). *P < .05; **P < .01; ***P < .001; ****P < .0001

To explore the effects of lncRNA NR2F1‐AS1 on breast cancer, we conducted Pearson's correlation analysis via GEPIA. We found that high expression of lncRNA NR2F1‐AS1 was correlated with high level of CD34 (*P* < .05, *R* = .34) and CD31 (*P* < .05, *R* = .30) in breast cancer, which were two vital markers of endothelial cells (Figure 1A,B). This indicated that lncRNA NR2F1‐AS1 might be associated with angiogenesis in breast cancer. Then, we overexpressed the expression of lncRNA NR2F1‐AS1 in HUVECs and in the meantime knock‐down of lncRNA NR2F1‐AS1 in HUVECs through lentivirus transfection and puromycin selection (Figure 1C). To explore the role of lncRNA NR2F1‐AS1 in angiogenesis, we conducted tube formation assay with above‐transfected HUVECs and found that HUVECs overexpressed with lncRNA NR2F1‐AS1 formed increased tubes and larger meshes than negative control cells. While after knocking down of lncRNA NR2F1‐AS1, the tube formation ability of HUVECs was decreased compared with the control (Figure 1D). This suggested that lncRNA NR2F1‐AS1 might play an important role in breast cancer angiogenesis.

### 3.2 lncRNA NR2F1‐AS1 promotes breast cancer angiogenesis in vitro

**FIGURE 2:**
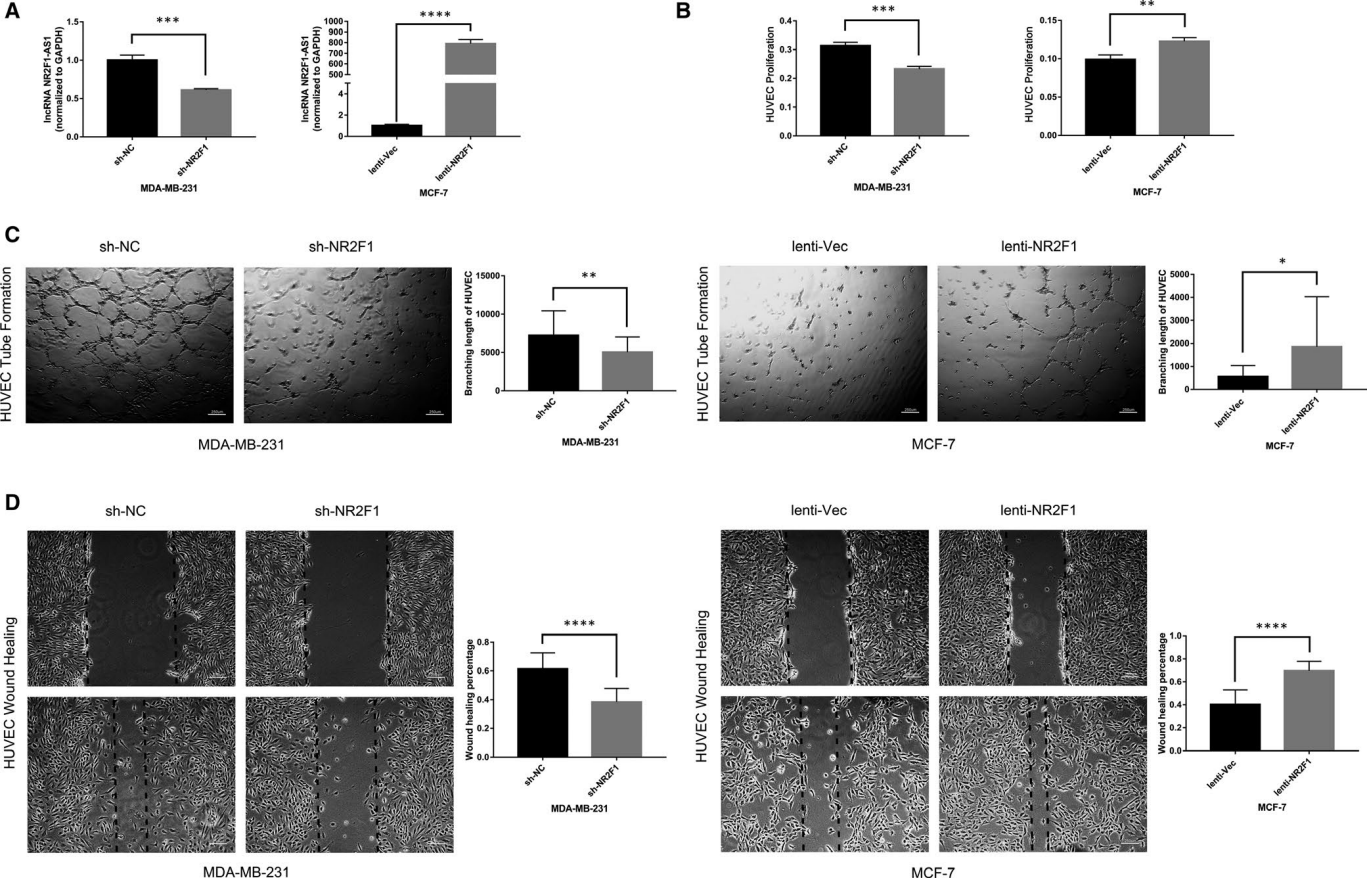
lncRNA NR2F1‐AS1 promotes breast cancer angiogenesis in vitro. A, Expression of lncRNA NR2F1‐AS1 in MDA‐MB‐231 cells knock‐down of lncRNA NR2F1‐AS1 and MCF‐7 cells overexpressing lncRNA NR2F1‐AS1. B, Proliferation of HUVECs at 48 h in the TCM from transfected MDA‐MB‐231 cells and at 96 h in the TCM from transfected MCF‐7 cells. C, Tube formation of HUVECs at 6 h in the TCM from transfected MDA‐MB‐231 and MCF‐7 cells (representative image). D, Wound healing of HUVECs at 48 h in the TCM from transfected MDA‐MB‐231 and MCF‐7 cells (representative image). *P < .05; **P < .01; ***P < .001; ****P < .0001

To verify the effects of lncRNA NR2F1‐AS1 on breast cancer angiogenesis, we first established the breast cancer cell line knock‐down of lncRNA NR2F1‐AS1 (MDA‐MB‐231‐sh‐NR2F1) compared with the negative control (MDA‐MB‐231‐sh‐NC), and overexpressing lncRNA NR2F1‐AS1 (MCF‐7‐lenti‐NR2F1) compared with the control group (MCF‐7‐lenti‐Vec) through lentivirus transfection (Figure 2A). Then, TCM was gathered from the transfected breast cancer cells for further proliferation assays, tube formation assays and wound healing assays with HUVECs. We found that TCM from MDA‐MB‐231‐sh‐NR2F1 cells induced lower proliferation rate of HUVECs than the TCM from MDA‐MB‐231‐sh‐NC cells did. In the meantime, the proliferation rate of HUVECs in the TCM from MCF‐7‐lenti‐NR2F1 cells was higher compared with that in the TCM from MCF‐7‐lenti‐Vec cells (Figure 2B). In addition, HUVECs in TCM from MDA‐MB‐231‐sh‐NR2F1 cells showed decreased tube formation ability compared with those in TCM of the control cells. However, increased tube structures were observed with HUVECs in TCM from MCF‐7‐lenti‐NR2F1 than those in TCM from MCF‐7‐lenti‐Vec cells (Figure 2C). Furthermore, the migration ability of HUVECs in TCM from MDA‐MB‐231‐sh‐NR2F1 cells was declined compared with that in TCM from MDA‐MB‐231‐sh‐NC cells. Besides, TCM from MCF‐7‐lenti‐NR2F1 cells enhanced the wound healing of HUVECs versus the TCM from MCF‐7‐lenti‐Vec cells (Figure 2D). Hence, lncRNA NR2F1‐AS1 could promote breast cancer angiogenesis in vitro.

### 3.3 lncRNA NR2F1‐AS1 promotes breast cancer angiogenesis in zebrafish model

**FIGURE 3:**
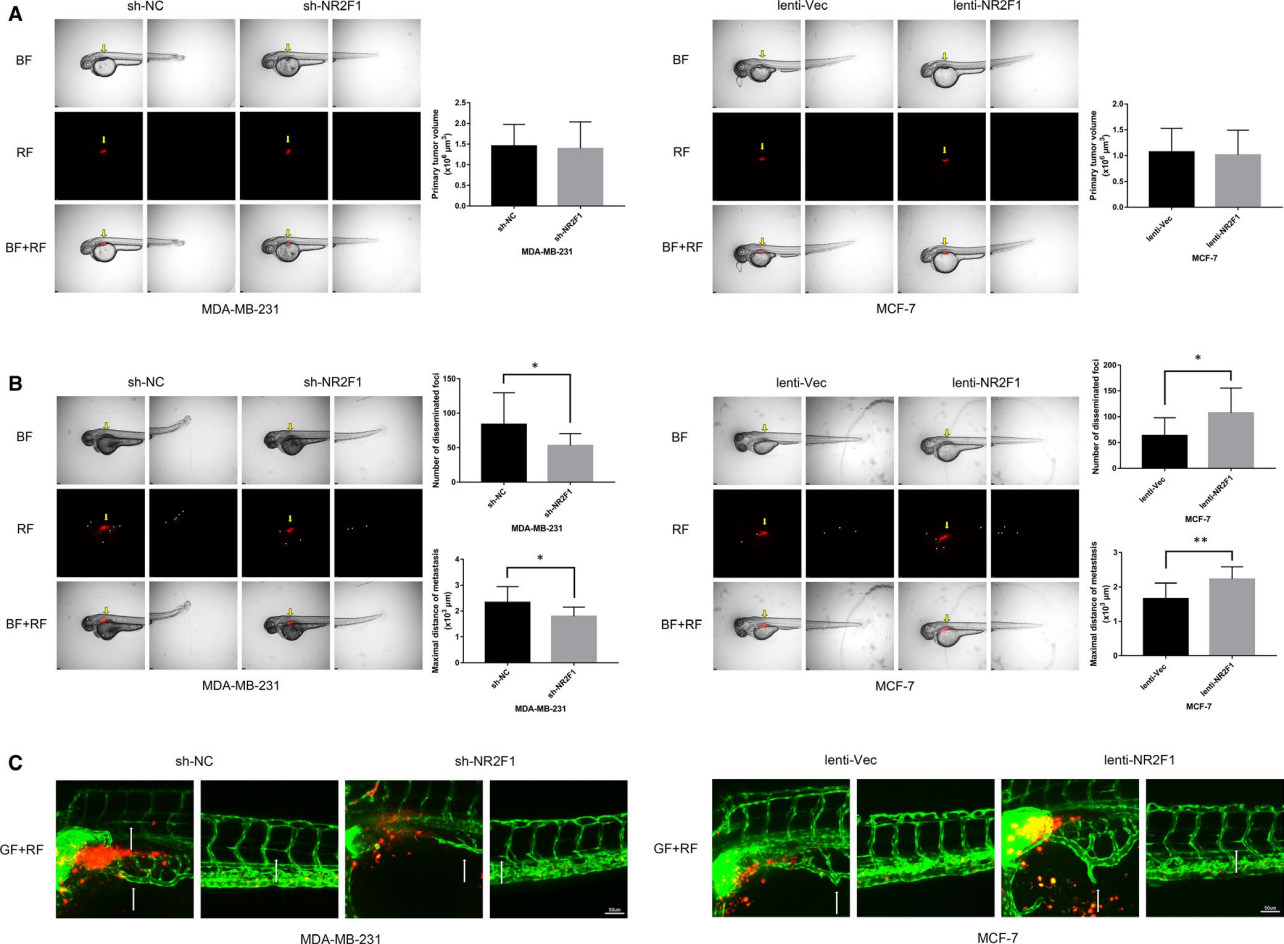
lncRNA NR2F1‐AS1 promotes breast cancer angiogenesis in zebrafish model. A, 0 h after microinjection of transfected MDA‐MB‐231 and MCF‐7 cells into the perivitelline space of embryos at 48 h post‐fertilization (representative image). B, Metastasis of the transfected breast cancer cells in zebrafish at 24 h post‐injection (representative image, for MDA‐MB‐231, n = 13/group, for MCF‐7, n = 14/group). C, Representative image of neo‐vascularization around breast cancer cells in zebrafish at 24 h post‐injection. Yellow arrows direct to the primary location of breast cancer cells. Small arrowheads direct to disseminated and metastatic tumour foci. White arrows direct to new formed tumour vascularization. BF, bright field; GF, green fluorescence; RF, red fluorescence. *P < .05; **P < .01; ***P < .001; ****P < .0001

To further demonstrate the relationship of lncRNA NR2F1‐AS1 and breast cancer angiogenesis in vivo, we labelled the above‐transfected breast cancer cells with Dil in red colour and microinjected the cells into the perivitelline space of zebrafish embryos at 48h post‐fertilization. The primary tumour sizes were similar in zebrafish embryos with MDA‐MB‐231‐sh‐NR2F1 cells and MDA‐MB‐231‐sh‐NC cells, as well as in those with MCF‐7‐lenti‐NR2F1 cells and MCF‐7‐lenti‐Vec cells (Figure 3A). At 24 hours after injection, the disseminated foci of MDA‐MB‐231‐sh‐NR2F1 cells were fewer and the maximal distances of metastasis of MDA‐MB‐231‐sh‐NR2F1 cells were shorter compared with that of MDA‐MB‐231‐sh‐NC cells (n = 13/group), while enhanced dissemination of tumour cells and longer distal metastasis were observed in zebrafish embryos implanted with MCF‐7‐lenti‐NR2F1 cells compared with those implanted with MCF‐7‐lenti‐Vec cells (n = 14/group) (Figure 3B). Furthermore, newly formed rugged tumour vessels were fewer in zebrafish embryos implanted with MDA‐MB‐231‐sh‐NR2F1 cells than those with MDA‐MB‐231‐sh‐NC cells. In the meantime, zebrafish embryos implanted with MCF‐7‐lenti‐NR2F1 cells presented improved neo‐angiogenesis versus zebrafish embryos with MCF‐7‐lenti‐Vec cells (Figure 3C). These results revealed that lncRNA NR2F1‐AS1 could promote breast cancer angiogenesis and metastasis in zebrafish model.

### 3.4 lncRNA NR2F1‐AS1 promotes breast cancer angiogenesis in mouse model

**FIGURE 4:**
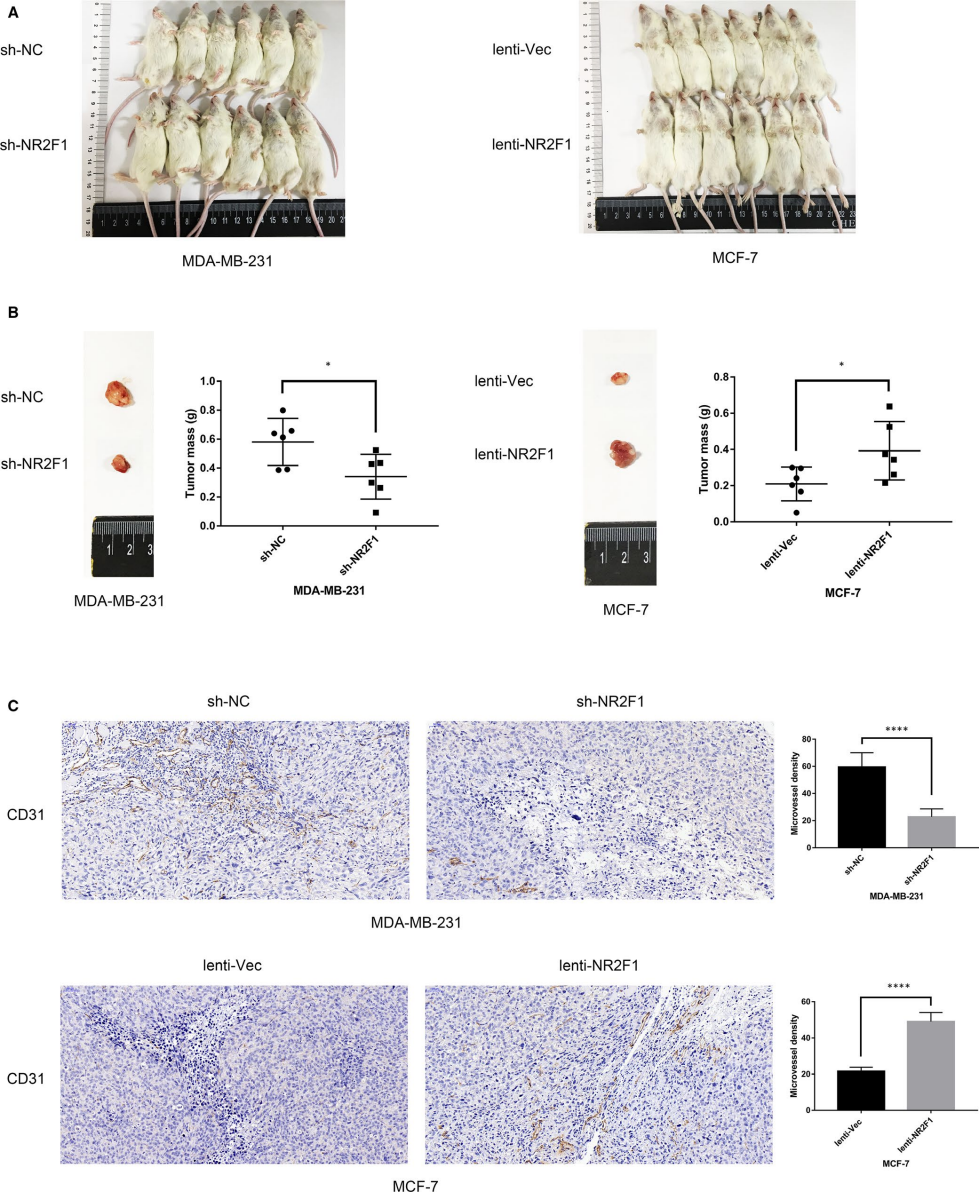
lncRNA NR2F1‐AS1 promotes breast cancer angiogenesis in mouse model. A, 28 d after implantation of transfected MDA‐MB‐231 and MCF‐7 cells in NOD/SCID mice (n = 6/group). B, Tumour size and weight at 28 d after implantation in mice (representative image). C, CD31 staining and MVD in tumour mass at 28 d after implantation in mice (representative image). *P < .05; **P < .01; ***P < .001; ****P < .0001

We then established the xenograft model to validate the angiogenetic effects of lncRNA NR2F1‐AS1 in mice. Transfected breast cancer cells were injected into the fat pad under the mammary glands of 4‐week‐old NOD/SCID mice (n = 6/group). The tumour masses were measured when the mice were sacrificed at 28 days after implantation (Figure 4A). Consistent with the results in zebrafish model, at 4 weeks after tumour cell implantation, the tumour masses of MDA‐MB‐231‐sh‐NR2F1 cells were smaller than those of MDA‐MB‐231‐sh‐NC cells (Figure 4B). And the tumour sizes of MCF‐7‐lenti‐NR2F1 cells were larger compared with the tumour sizes of MCF7‐lenti‐Vec cells in mice (Figure 4B). Moreover, we examined the expression of CD31 in the tumour masses through IHC staining to detect the tumour angiogenesis. Compared with MDA‐MB‐231‐sh‐NC–derived tumours, the tumour masses of MDA‐MB‐231‐sh‐NR2F1 cells displayed smaller and fewer tumour vessels, and the MVD was significantly lower. Meanwhile, the CD31‐marked tumour vessels in MCF‐7‐lenti‐NR2F1–derived tumours were larger and more, while the MVD was obviously higher compared with that of MCF‐7‐lenti‐Vec–derived tumours (Figure 4C). Therefore, lncRNA NR2F1‐AS1 could promote breast cancer angiogenesis and growth in mouse model.

### 3.5 lncRNA NR2F1‐AS1 promotes breast cancer angiogenesis via IGF‐1/IGF‐1R/ERK pathway

**FIGURE 5:**
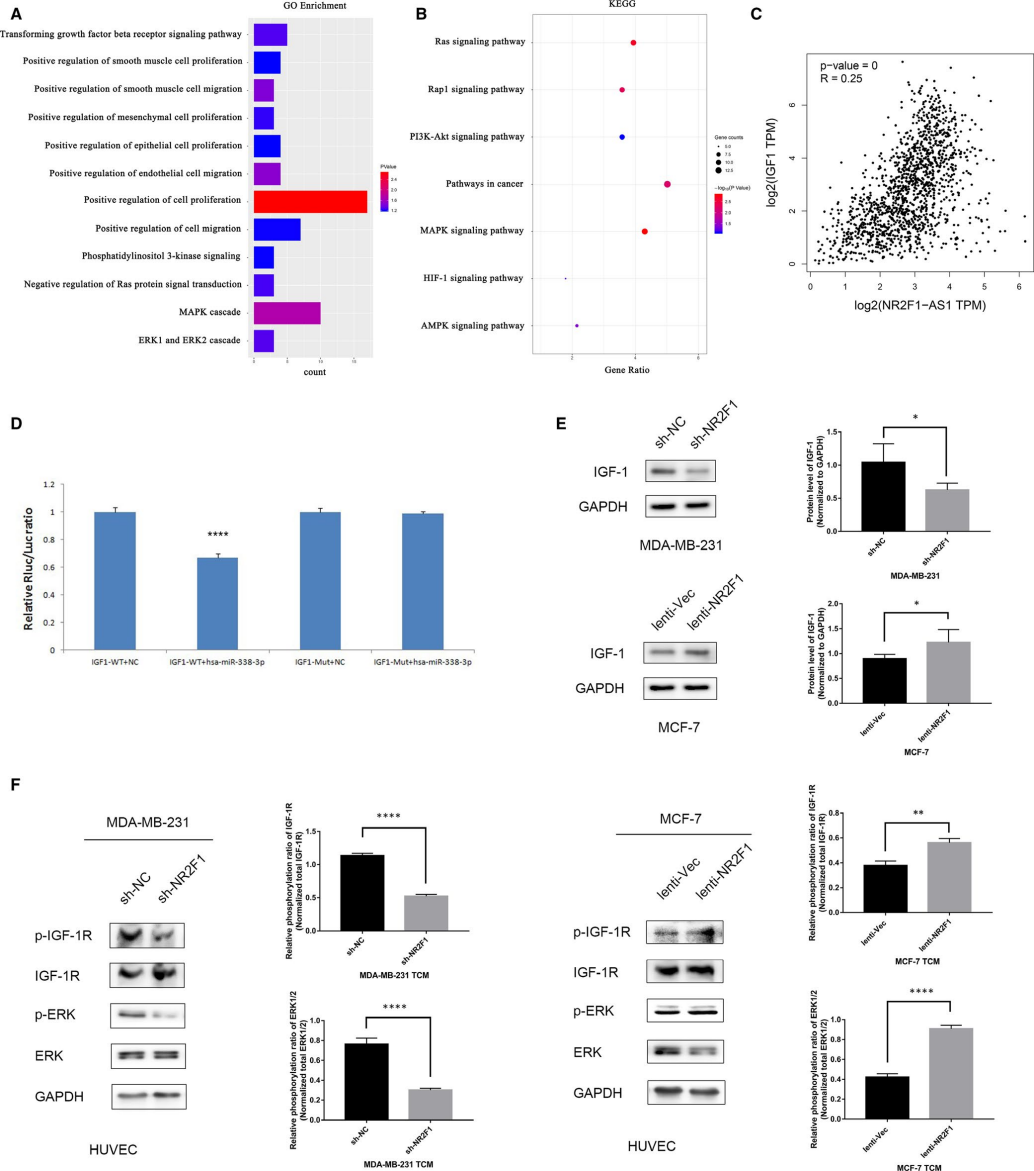
lncRNA NR2F1‐AS1 promotes breast cancer angiogenesis via IGF‐1/IGF‐1R/ERK pathway. A, GO enrichment of the target genes of miRNA‐338‐3p. B, KEGG analysis of the target genes of miRNA‐338‐3p. C, Pearson's correlation analysis of lncRNA NR2F1‐AS1 and IGF‐1. D, Dual‐luciferase reporter gene assay of miRNA‐338‐3p and IGF‐1. E, Protein expression of IGF‐1 in transfected MDA‐MB‐231 and MCF‐7 cells (representative image) and the Western blot analysis. F, Phosphorylation of IGF‐1R and ERK1/2 in HUVECs cultivated in TCM from transfected breast cancer cells (representative image) and the Western blot analysis. *P < .05; **P < .01; ***P < .001; ****P < .0001

It is reported that lncRNA NR2F1‐AS1 could directly target miRNA‐338‐3p to exert tumour‐promoting effects by dual‐luciferase reporter analysis and RNA immunoprecipitation analysis. In that dual‐luciferase reporter assay, cotransfecting of miRNA‐338‐3p mimics and NR2F1‐AS1‐wt led to weaker luciferase activity nevertheless NR2F1‐AS1‐mut transfecting resulted in no significance. In that RNA immunoprecipitation assay, both lncRNA NR2F1‐AS1 and miRNA‐338‐3p were enriched in Ago protein compared with IgG.^21^ Thus in breast cancer, miRNA‐338‐3p might be the downstream binding miRNA of lncRNA NR2F1‐AS1. As miRNA‐338‐3p was found to promote angiogenesis in hepatocellular carcinoma,^32^ it is likely that miRNA‐338‐3p regulated the angiogenetic effects of lncRNA NR2F1‐AS1 in breast cancer. To verify the function of miRNA‐338‐3p on breast cancer angiogenesis, we then predicted the downstream target genes of miRNA‐338‐3p using TargetScan software and MiRanda software, respectively, collected the intersection of the predicted genes (Table S1) and then conducted GO enrichment (Figure 5A) and KEGG analysis (Figure 5B). We found that the target genes of miRNA‐338‐3p were closely related to tumour angiogenesis. Therefore, lncRNA NR2F1‐AS1 might promote breast cancer angiogenesis through sponging miRNA‐338‐3p.

Among the predicted target genes of miRNA‐338‐3p, we found that IGF‐1 might be closely relevant to the effects of lncRNA NR2F1‐AS1 through Pearson's correlation analysis (Figure 5C), and the predicted binding sites between miRNA‐338‐3p and IGF‐1 through TargetScan software and miRanda software were shown in Tables 1 and 2, respectively. Then, we conducted dual‐luciferase reporter gene assay and found that miRNA‐338‐3p transfection reduced the luciferase activities of wild‐type IGF‐1 while the luciferase activities of mutated‐type IGF‐1 maintained the same level after transfecting with miRNA‐338‐3p (Figure 5D). This validated that miRNA‐338‐3p could directly bind to the 3'‐UTR of IGF‐1 to regulate the expression of IGF‐1. To further consolidate the role of IGF‐1 in the angiogenetic effects of lncRNA NR2F1‐AS1, we then examined the protein expression of IGF‐1 in transfected breast cancer cells. IGF‐1 expression was decreased in MDA‐MB‐231‐sh‐NR2F1 cells than that in MDA‐MB‐231‐sh‐NC cells. And in MCF‐7‐lenti‐NR2F1 cells, the protein expression of IGF‐1 was higher than that in the control cells (Figure 5E). Hence, lncRNA NR2F1‐AS1 might promote breast cancer angiogenesis through inducing IGF‐1 expression by sponging miRNA‐338‐3p.

**TABLE 1:** Predicted binding sites between miR‐338‐3p and IGF‐1 through TargetScan

	Predicted consequential pairing of target region (top) and miRNA (bottom)	Site type	Context++ score	Context++ score percentile	Weighted context++ score	Conserved branch length	*P*_CT_
Position 362‐368 of IGF1 3' UTR	5'…UCUCUGAAUCUUGGC*UGCUGG*AG…	7mer‐A1	−0.07	79	−0.07	3.756	.30
	|||| |||||						
hsa‐miR‐338‐3p	3' GUUGUUUUAGUGACU*ACGACC*U						

**TABLE 2:** Predicted binding sites between miR‐338‐3p and IGF‐1 through MiRanda

	Predicted consequential pairing of target region (top) and miRNA (bottom)	Conservation	Align score	Seed cat	Energy	MirSVR score
Position 347‐368 of IGF1 3' UTR	5' ucuCUGAAUCUUGGCUGCUGGa	0.6935	131	79	−18.25	−0.0002
	|:||||: | ||||||					
hsa‐miR‐338‐3p	3' guuGUUUUAGUGACUACGACCu					

IGF‐1 was reported to combine with and then phosphorylate IGF‐1R, which could further promote cell proliferation, differentiation and metabolism.^33^ Besides, the secreted IGF‐1 binding to IGF‐1R in vascular endothelial cells could inhibit endothelial cell apoptosis and promote endothelial cell proliferation, migration and angiogenesis.^34^ Thus, we detected the phosphorylation of IGF‐1R in HUVECs after TCM culturing and found that the MDA‐MB‐231‐sh‐NR2F1 TCM induced weaker phosphorylation of IGF‐1R in HUVECs than MDA‐MB‐231‐sh‐NC TCM, while the phosphorylated IGF‐1R protein was increased in HUVECs from MCF‐7‐lenti‐NR2F1 TCM compared with that from control TCM. Hence, lncRNA NR2F1‐AS1 induced IGF‐1 in breast cancer cells could activate the phosphorylation of IGF‐1R in HUVECs, which then took effects on angiogenesis. Researchers have found the phosphorylation of IGF‐1R led to activation of extracellular signal‐regulated kinase (ERK) pathway,^35^ which was not only essential for cell growth but also vital for angiogenesis.^36^, ^37^ Thus we further examined the phosphorylation of ERK pathway in HUVECs cultured with TCM. We found that after culturing in TCM from MDA‐MB‐231‐sh‐NR2F1 cells, the phosphorylation of ERK1/2 in HUVECs was declined compared with that in TCM from MDA‐MB‐231‐sh‐NC cells. And MCF‐7‐lenti‐NR2F1 TCM enhanced the phosphorylation of ERK1/2 in HUVECs versus MCF‐7‐lenti‐Vec TCM (Figure 5F). These results were consistent with the consequences of phosphorylation of IGF‐1R, hinting that the activation of IGF‐1R further activated ERK pathway to promote angiogenesis. Thus, lncRNA NR2F1‐AS1 might promote breast cancer angiogenesis through activating IGF‐1/IGF‐1R/ERK pathway.

## 4 DISCUSSION

Tumour angiogenesis is an important procedure for tumour growth and metastasis.^1^, ^2^ In our study, we found that lncRNA NR2F1‐AS1 was positively related to the vessel target CD31 and CD34 in breast cancer, and could enhance the tube formation ability of HUVECs in vitro, which hinted the potential role of lncRNA NR2F1‐AS1 in breast cancer angiogenesis. In breast cancer cell, lncRNA NR2F1‐AS1 improved the TCM induced proliferation, tube formation and migration ability of HUVECs. Also, in zebrafish model, lncRNA NR2F1‐AS1 facilitated the neovascularization due to breast cancer cell. And lncRNA NR2F1‐AS1 also promoted the metastasis of breast cancer cells in zebrafish, which might probably result from the lncRNA NR2F1‐AS1 increased tumour vasculature. In mouse model, lncRNA NR2F1‐AS1 induced more tumour vessels and higher MVD in the tumour mass. Besides, lncRNA NR2F1‐AS1 also facilitated breast cancer xenograft growth in mice and the increased vascularization might be the major cause of that pro‐growth effects of lncRNA‐NR2F1. These results revealed that lncRNA NR2F1‐AS1 could promote breast cancer angiogenesis both in vitro and in vivo, and the pro‐angiogenesis effects could further accelerate breast cancer growth and metastasis.

lncRNAs are regarded to play essential parts in various cancer‐related procedure,^12^, ^13^, ^14^, ^15^, ^16^ during which sponging miRNA to protect downstream targets is one of the major mechanism.^17^, ^18^ lncRNA NR2F1‐AS1 could promote drug resistance in hepatocellular carcinoma^19^ and was related with progression in endometrial cancer.^20^ In thyroid cancer, lncRNA NR2F1‐AS1 promoted cell proliferation and migration yet suppress apoptosis through directly binding to miRNA‐338‐3p,^21^ while down‐regulation of miRNA‐338‐3p promoted angiogenesis in hepatocellular carcinoma.^32^ In breast cancer, we found miRNA‐338‐3p, as the target binding miRNA of lncRNA NR2F1‐AS1, could probably exert angiogenetic effects according to bioinformatics prediction, which was consistent with the results in hepatocellular carcinoma. Thus, lncRNA NR2F1‐AS1 induced breast cancer angiogenesis probably through sponging miRNA‐338‐3p.

**FIGURE 6:**
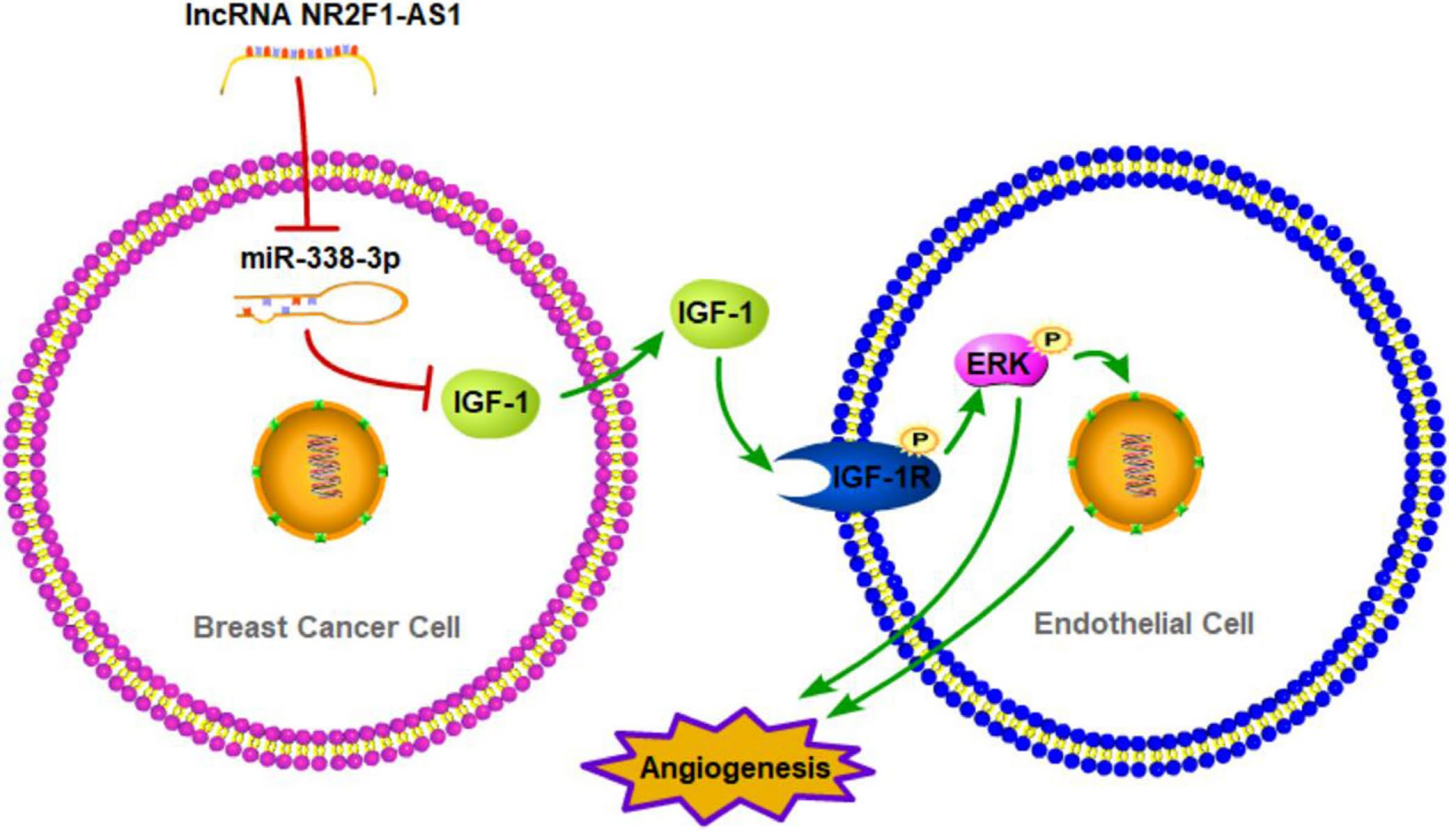
Schematic diagram of lncRNA NR2F1‐AS1 promoting breast cancer angiogenesis through IGF‐1/IGF‐1R/ERK pathway via sponging miRNA‐338‐3p

Among the target genes of miRNA‐338‐3p, we verified that IGF‐1, the most promising target on the basis of prediction, could directly bind to miRNA‐338‐3p. In the meantime, the expression of IGF‐1 could be positively regulated by lncRNA NR2F1‐AS1, which was the binding competing endogenous RNA (ceRNA) of miRNA‐338‐3p. Hence, lncRNA NR2F1‐AS1 could sponge miRNA‐338‐3p to protect IGF‐1 from suppression. It is reported that secreted IGF‐1 could combine with IGF‐1R in endothelial cells to promote angiogenesis,^34^ and the activation of IGF‐1R could further activate ERK pathway.^35^ In our study, lncRNA NR2F1‐AS1 induced expression of IGF‐1 in breast cancer cells, which then increased the phosphorylation of IGF‐1R and ERK1/2 in HUVECs. Hence, lncRNA NR2F1‐AS1 could promote breast cancer angiogenesis via IGF‐1/IGF‐1R/ERK pathway through sponging miRNA‐338‐3p (Figure 6).

In conclusion, we revealed the potential angiogenetic effects of lncRNA NR2F1‐AS1 in breast cancer. lncRNA NR2F1‐AS1 promoted breast cancer angiogenesis both in vitro and in vivo. The underlying mechanism was uncovered that lncRNA NR2F1 sponged miRNA‐338‐3p to induce IGF‐1 in breast cancer cells, which further activated IGF‐1R and ERK pathway in HUVECs. Thus, lncRNA NR2F1‐AS1 could be a new promising therapy target in breast cancer.

## CONFLICT OF INTEREST

Authors declare no conflicts of interest for this article.

## AUTHOR CONTRIBUTIONS

**Qi Zhang:** Data curation (equal); Formal analysis (equal); Investigation (equal); Methodology (equal); Visualization (lead); Writing‐original draft (lead); Writing‐review & editing (lead). **Tianfu Li:** Data curation (equal); Formal analysis (supporting); Investigation (supporting); Methodology (supporting); Software (lead); Visualization (equal). **Zhecun Wang:** Conceptualization (supporting); Data curation (equal); Formal analysis (equal); Investigation (equal); Methodology (equal); Resources (supporting); Software (supporting). **Xiaying Kuang:** Conceptualization (supporting); Funding acquisition (equal); Project administration (equal); Resources (lead). **Nan Shao:** Conceptualization (equal); Funding acquisition (equal); Project administration (equal); Resources (supporting); Supervision (equal); Validation (equal). **Ying Lin:** Conceptualization (equal); Funding acquisition (lead); Project administration (lead); Resources (equal); Supervision (lead); Validation (lead).

## ETHICAL APPROVAL

The approval for zebrafish experiments was obtained from Sun Yat‐sen University Animal Care and Use Committee of the Zebrafish Model Animal Facility, Institute of Clinical and Translational Research, Sun Yat‐sen University.

The approval for mice experiments was obtained from the Animal Care and Use Committee of the First Affiliated Hospital of Sun Yat‐sen University.

## Supporting information

Table S1

## Data Availability

The data that support the findings of this study are available from the corresponding author upon reasonable request.
