# Supplementary material for: lncRNA NR2F1‐AS1 promotes breast cancer angiogenesis through activating IGF‐1/IGF‐1R/ERK pathway
Source: J Cell Mol Med. 2020 Jun 17;24(14):8236–47. doi: 10.1111/jcmm.15499 (PMC7348140; doi:10.1111/jcmm.15499)
Supplement: Supplementary file 1 — Table S1 [file JCMM-24-8236-s001.docx]

**Supporting Table.** Intersection of predicted target genes of miRNA-338-3p by TargetScan and MiRanda.

| No. | Target gene | Representative transcript | Gene name |
| --- | --- | --- | --- |
| 1 | UBE2Q1 | ENST00000292211.4 | ubiquitin-conjugating enzyme E2Q family member 1 |
| 2 | WNK4 | ENST00000246914.5 | WNK lysine deficient protein kinase 4 |
| 3 | PPP1R1A | ENST00000257905.8 | protein phosphatase 1, regulatory (inhibitor) subunit 1A |
| 4 | GNG12 | ENST00000370982.3 | guanine nucleotide binding protein (G protein), gamma 12 |
| 5 | THBS1 | ENST00000260356.5 | thrombospondin 1 |
| 6 | TM6SF1 | ENST00000379390.6 | transmembrane 6 superfamily member 1 |
| 7 | MED11 | ENST00000575284.1 | mediator complex subunit 11 |
| 8 | COX4I1 | ENST00000564903.1 | cytochrome c oxidase subunit IV isoform 1 |
| 9 | TRIM33 | ENST00000358465.2 | tripartite motif containing 33 |
| 10 | MTHFD1L | ENST00000367321.3 | methylenetetrahydrofolate dehydrogenase (NADP+ dependent) 1-like |
| 11 | DES | ENST00000373960.3 | desmin |
| 12 | ICOS | ENST00000435193.1 | inducible T-cell co-stimulator |
| 13 | ETS1 | ENST00000531611.1 | v-ets avian erythroblastosis virus E26 oncogene homolog 1 |
| 14 | ZBTB39 | ENST00000300101.2 | zinc finger and BTB domain containing 39 |
| 15 | RAB30 | ENST00000533486.1 | RAB30, member RAS oncogene family |
| 16 | PDIK1L | ENST00000374271.4 | PDLIM1 interacting kinase 1 like |
| 17 | HOXA3 | ENST00000396352.4 | homeobox A3 |
| 18 | TERF2 | ENST00000254942.3 | telomeric repeat binding factor 2 |
| 19 | LARP4 | ENST00000398473.2 | La ribonucleoprotein domain family, member 4 |
| 20 | PABPC1L | ENST00000537323.1 | poly(A) binding protein, cytoplasmic 1-like |
| 21 | TAF1 | ENST00000373790.4 | TAF1 RNA polymerase II, TATA box binding protein (TBP)-associated factor, 250kDa |
| 22 | NUFIP2 | ENST00000225388.4 | nuclear fragile X mental retardation protein interacting protein 2 |
| 23 | WAPAL | ENST00000298767.5 | wings apart-like homolog (Drosophila) |
| 24 | RPP25 | ENST00000322177.5 | ribonuclease P/MRP 25kDa subunit |
| 25 | RASA1 | ENST00000456692.2 | RAS p21 protein activator (GTPase activating protein) 1 |
| 26 | LDOC1L | ENST00000341255.3 | leucine zipper, down-regulated in cancer 1-like |
| 27 | ARHGEF10L | ENST00000452522.1 | Rho guanine nucleotide exchange factor (GEF) 10-like |
| 28 | GPD2 | ENST00000540309.1 | glycerol-3-phosphate dehydrogenase 2 (mitochondrial) |
| 29 | SEC61A2 | ENST00000379020.4 | Sec61 alpha 2 subunit (S. cerevisiae) |
| 30 | HUNK | ENST00000270112.2 | hormonally up-regulated Neu-associated kinase |
| 31 | MAFB | ENST00000373313.2 | v-maf avian musculoaponeurotic fibrosarcoma oncogene homolog B |
| 32 | RAB14 | ENST00000373840.4 | RAB14, member RAS oncogene family |
| 33 | ARPC1B | ENST00000252725.5 | actin related protein 2/3 complex, subunit 1B, 41kDa |
| 34 | VPS52 | ENST00000482399.1 | vacuolar protein sorting 52 homolog (S. cerevisiae) |
| 35 | MAPK1IP1L | ENST00000395468.4 | mitogen-activated protein kinase 1 interacting protein 1-like |
| 36 | RNF114 | ENST00000244061.2 | ring finger protein 114 |
| 37 | NRP1 | ENST00000374875.1 | neuropilin 1 |
| 38 | SLCO3A1 | ENST00000318445.6 | solute carrier organic anion transporter family, member 3A1 |
| 39 | CHL1 | ENST00000256509.2 | cell adhesion molecule L1-like |
| 40 | SPHAR | ENST00000366688.3 | S-phase response (cyclin related) |
| 41 | IPO9 | ENST00000361565.4 | importin 9 |
| 42 | MBD5 | ENST00000407073.1 | methyl-CpG binding domain protein 5 |
| 43 | MYT1L | ENST00000399161.2 | myelin transcription factor 1-like |
| 44 | FRZB | ENST00000295113.4 | frizzled-related protein |
| 45 | SEPT8 | ENST00000378706.1 | septin 8 |
| 46 | CACNB4 | ENST00000539935.1 | calcium channel, voltage-dependent, beta 4 subunit |
| 47 | B4GALT7 | ENST00000029410.5 | xylosylprotein beta 1,4-galactosyltransferase, polypeptide 7 |
| 48 | SULT4A1 | ENST00000330884.4 | sulfotransferase family 4A, member 1 |
| 49 | PANK1 | ENST00000322191.6 | pantothenate kinase 1 |
| 50 | FBXW7 | ENST00000281708.4 | F-box and WD repeat domain containing 7, E3 ubiquitin protein ligase |
| 51 | RASSF4 | ENST00000374417.2 | Ras association (RalGDS/AF-6) domain family member 4 |
| 52 | FAM120A | ENST00000333936.5 | family with sequence similarity 120A |
| 53 | MACROD2 | ENST00000217246.4 | MACRO domain containing 2 |
| 54 | PTPN12 | ENST00000248594.6 | protein tyrosine phosphatase, non-receptor type 12 |
| 55 | ZSCAN12 | ENST00000361028.1 | zinc finger and SCAN domain containing 12 |
| 56 | RBBP5 | ENST00000264515.6 | retinoblastoma binding protein 5 |
| 57 | MANF | ENST00000528157.1 | mesencephalic astrocyte-derived neurotrophic factor |
| 58 | ZDHHC18 | ENST00000374142.4 | zinc finger, DHHC-type containing 18 |
| 59 | PTPRT | ENST00000373187.1 | protein tyrosine phosphatase, receptor type, T |
| 60 | XRN1 | ENST00000264951.4 | 5'-3' exoribonuclease 1 |
| 61 | SCAI | ENST00000336505.6 | suppressor of cancer cell invasion |
| 62 | TTL | ENST00000233336.6 | tubulin tyrosine ligase |
| 63 | CELSR2 | ENST00000271332.3 | cadherin, EGF LAG seven-pass G-type receptor 2 |
| 64 | F10 | ENST00000375551.3 | coagulation factor X |
| 65 | SNX18 | ENST00000343017.6 | sorting nexin 18 |
| 66 | RGS7BP | ENST00000334025.2 | regulator of G-protein signaling 7 binding protein |
| 67 | MAF | ENST00000393350.1 | v-maf avian musculoaponeurotic fibrosarcoma oncogene homolog |
| 68 | PELI3 | ENST00000320740.7 | pellino E3 ubiquitin protein ligase family member 3 |
| 69 | NHS | ENST00000380060.3 | Nance-Horan syndrome (congenital cataracts and dental anomalies) |
| 70 | AKAP12 | ENST00000402676.2 | A kinase (PRKA) anchor protein 12 |
| 71 | PCGF3 | ENST00000362003.5 | polycomb group ring finger 3 |
| 72 | F11R | ENST00000368026.6 | F11 receptor |
| 73 | ATXN7L1 | ENST00000419735.3 | ataxin 7-like 1 |
| 74 | UBE2G1 | ENST00000396981.2 | ubiquitin-conjugating enzyme E2G 1 |
| 75 | DGKH | ENST00000261491.5 | diacylglycerol kinase, eta |
| 76 | ZBTB43 | ENST00000449886.1 | zinc finger and BTB domain containing 43 |
| 77 | BBX | ENST00000415149.2 | bobby sox homolog (Drosophila) |
| 78 | YRDC | ENST00000373044.2 | yrdC N(6)-threonylcarbamoyltransferase domain containing |
| 79 | SLK | ENST00000335753.4 | STE20-like kinase |
| 80 | ARL5A | ENST00000295087.8 | ADP-ribosylation factor-like 5A |
| 81 | SNAP29 | ENST00000215730.7 | synaptosomal-associated protein, 29kDa |
| 82 | TPM3 | ENST00000368531.2 | tropomyosin 3 |
| 83 | G6PC2 | ENST00000421979.1 | glucose-6-phosphatase, catalytic, 2 |
| 84 | NFIA | ENST00000403491.3 | nuclear factor I/A |
| 85 | TACC1 | ENST00000330691.6 | transforming, acidic coiled-coil containing protein 1 |
| 86 | TSPYL1 | ENST00000368608.3 | TSPY-like 1 |
| 87 | CDH1 | ENST00000261769.5 | cadherin 1, type 1, E-cadherin (epithelial) |
| 88 | SEMA6D | ENST00000355997.3 | sema domain, transmembrane domain (TM), and cytoplasmic domain, (semaphorin) 6D |
| 89 | RUNX2 | ENST00000371432.3 | runt-related transcription factor 2 |
| 90 | TGOLN2 | ENST00000377386.3 | trans-golgi network protein 2 |
| 91 | TSFM | ENST00000540550.1 | Ts translation elongation factor, mitochondrial |
| 92 | HCN1 | ENST00000303230.4 | hyperpolarization activated cyclic nucleotide-gated potassium channel 1 |
| 93 | PCDH1 | ENST00000503492.1 | protocadherin 1 |
| 94 | FAM73B | ENST00000277475.5 | family with sequence similarity 73, member B |
| 95 | PPP4R1 | ENST00000400556.3 | protein phosphatase 4, regulatory subunit 1 |
| 96 | MSN | ENST00000360270.5 | moesin |
| 97 | UBFD1 | ENST00000395878.3 | ubiquitin family domain containing 1 |
| 98 | RASAL1 | ENST00000546530.1 | RAS protein activator like 1 (GAP1 like) |
| 99 | SEPT4 | ENST00000426861.1 | septin 4 |
| 100 | TSPYL4 | ENST00000420283.1 | TSPY-like 4 |
| 101 | TBL1XR1 | ENST00000430069.1 | transducin (beta)-like 1 X-linked receptor 1 |
| 102 | PRDM15 | ENST00000422911.1 | PR domain containing 15 |
| 103 | FTCD | ENST00000355384.2 | formimidoyltransferase cyclodeaminase |
| 104 | ALKBH6 | ENST00000486389.1 | alkB, alkylation repair homolog 6 (E. coli) |
| 105 | MRRF | ENST00000344641.3 | mitochondrial ribosome recycling factor |
| 106 | MAPK1 | ENST00000215832.6 | mitogen-activated protein kinase 1 |
| 107 | ADORA1 | ENST00000367235.1 | adenosine A1 receptor |
| 108 | USP45 | ENST00000392738.2 | ubiquitin specific peptidase 45 |
| 109 | RRM1 | ENST00000300738.5 | ribonucleotide reductase M1 |
| 110 | POLR3C | ENST00000369294.1 | polymerase (RNA) III (DNA directed) polypeptide C (62kD) |
| 111 | TEX19 | ENST00000333437.4 | testis expressed 19 |
| 112 | DBNL | ENST00000494774.1 | drebrin-like |
| 113 | SLC44A1 | ENST00000374720.3 | solute carrier family 44 (choline transporter), member 1 |
| 114 | SOX4 | ENST00000244745.1 | SRY (sex determining region Y)-box 4 |
| 115 | UPK1B | ENST00000264234.3 | uroplakin 1B |
| 116 | PIRT | ENST00000580256.2 | phosphoinositide-interacting regulator of transient receptor potential channels |
| 117 | CBL | ENST00000264033.4 | Cbl proto-oncogene, E3 ubiquitin protein ligase |
| 118 | HIF1AN | ENST00000299163.6 | hypoxia inducible factor 1, alpha subunit inhibitor |
| 119 | FAM58A | ENST00000406277.2 | family with sequence similarity 58, member A |
| 120 | ZFAND3 | ENST00000287218.4 | zinc finger, AN1-type domain 3 |
| 121 | ZNF607 | ENST00000355202.4 | zinc finger protein 607 |
| 122 | USP7 | ENST00000344836.4 | ubiquitin specific peptidase 7 (herpes virus-associated) |
| 123 | NKX2-1 | ENST00000354822.5 | NK2 homeobox 1 |
| 124 | AAK1 | ENST00000409085.4 | AP2 associated kinase 1 |
| 125 | DUSP2 | ENST00000288943.4 | dual specificity phosphatase 2 |
| 126 | SS18L1 | ENST00000331758.3 | synovial sarcoma translocation gene on chromosome 18-like 1 |
| 127 | ALPK3 | ENST00000258888.5 | alpha-kinase 3 |
| 128 | CAMK2G | ENST00000351293.3 | calcium/calmodulin-dependent protein kinase II gamma |
| 129 | SNX30 | ENST00000374232.3 | sorting nexin family member 30 |
| 130 | TNRC6B | ENST00000335727.9 | trinucleotide repeat containing 6B |
| 131 | TMEM92 | ENST00000300433.3 | transmembrane protein 92 |
| 132 | EIF4E3 | ENST00000425534.3 | eukaryotic translation initiation factor 4E family member 3 |
| 133 | PLA2G15 | ENST00000566188.1 | phospholipase A2, group XV |
| 134 | ZNF207 | ENST00000394670.4 | zinc finger protein 207 |
| 135 | C14orf1 | ENST00000256319.6 | chromosome 14 open reading frame 1 |
| 136 | BRPF3 | ENST00000534400.1 | bromodomain and PHD finger containing, 3 |
| 137 | PCBD2 | ENST00000512783.1 | pterin-4 alpha-carbinolamine dehydratase/dimerization cofactor of hepatocyte nuclear factor 1 alpha (TCF1) 2 |
| 138 | CAMK2A | ENST00000348628.6 | calcium/calmodulin-dependent protein kinase II alpha |
| 139 | ZNF579 | ENST00000325421.4 | zinc finger protein 579 |
| 140 | DLG2 | ENST00000398309.2 | discs, large homolog 2 (Drosophila) |
| 141 | AGPAT5 | ENST00000285518.6 | 1-acylglycerol-3-phosphate O-acyltransferase 5 |
| 142 | STYX | ENST00000354586.4 | serine/threonine/tyrosine interacting protein |
| 143 | C10orf54 | ENST00000394957.3 | chromosome 10 open reading frame 54 |
| 144 | ARMCX3 | ENST00000341189.4 | armadillo repeat containing, X-linked 3 |
| 145 | TSPYL5 | ENST00000322128.3 | TSPY-like 5 |
| 146 | SRI | ENST00000265729.2 | sorcin |
| 147 | CNOT6 | ENST00000393356.1 | CCR4-NOT transcription complex, subunit 6 |
| 148 | ACTR2 | ENST00000260641.5 | ARP2 actin-related protein 2 homolog (yeast) |
| 149 | SCARF1 | ENST00000571272.1 | scavenger receptor class F, member 1 |
| 150 | TRIM73 | ENST00000430211.1 | tripartite motif containing 73 |
| 151 | LRRC59 | ENST00000225972.7 | leucine rich repeat containing 59 |
| 152 | SYT2 | ENST00000367267.1 | synaptotagmin II |
| 153 | DUSP16 | ENST00000298573.4 | dual specificity phosphatase 16 |
| 154 | ADAM17 | ENST00000310823.3 | ADAM metallopeptidase domain 17 |
| 155 | DALRD3 | ENST00000441576.2 | DALR anticodon binding domain containing 3 |
| 156 | SEC31B | ENST00000370345.3 | SEC31 homolog B (S. cerevisiae) |
| 157 | TMEM33 | ENST00000504986.1 | transmembrane protein 33 |
| 158 | FBXO33 | ENST00000298097.7 | F-box protein 33 |
| 159 | PBX1 | ENST00000367897.1 | pre-B-cell leukemia homeobox 1 |
| 160 | TANC1 | ENST00000263635.6 | tetratricopeptide repeat, ankyrin repeat and coiled-coil containing 1 |
| 161 | FGFR2 | ENST00000369061.4 | fibroblast growth factor receptor 2 |
| 162 | PEX5L | ENST00000467460.1 | peroxisomal biogenesis factor 5-like |
| 163 | FMOD | ENST00000354955.4 | fibromodulin |
| 164 | RNF141 | ENST00000265981.2 | ring finger protein 141 |
| 165 | BRD4 | ENST00000263377.2 | bromodomain containing 4 |
| 166 | BAMBI | ENST00000375533.3 | BMP and activin membrane-bound inhibitor |
| 167 | KIF2A | ENST00000381103.2 | kinesin heavy chain member 2A |
| 168 | TXLNA | ENST00000373610.3 | taxilin alpha |
| 169 | CLCN6 | ENST00000312413.6 | chloride channel, voltage-sensitive 6 |
| 170 | DCUN1D3 | ENST00000324344.4 | DCN1, defective in cullin neddylation 1, domain containing 3 |
| 171 | CRELD1 | ENST00000326434.5 | cysteine-rich with EGF-like domains 1 |
| 172 | AP4E1 | ENST00000261842.5 | adaptor-related protein complex 4, epsilon 1 subunit |
| 173 | OSBPL3 | ENST00000313367.2 | oxysterol binding protein-like 3 |
| 174 | INSM1 | ENST00000310227.1 | insulinoma-associated 1 |
| 175 | TAS1R1 | ENST00000328191.4 | taste receptor, type 1, member 1 |
| 176 | HPSE2 | ENST00000370546.1 | heparanase 2 |
| 177 | SV2A | ENST00000369146.3 | synaptic vesicle glycoprotein 2A |
| 178 | TUBB6 | ENST00000591208.1 | tubulin, beta 6 class V |
| 179 | CRK | ENST00000398970.5 | v-crk avian sarcoma virus CT10 oncogene homolog |
| 180 | HAPLN1 | ENST00000274341.4 | hyaluronan and proteoglycan link protein 1 |
| 181 | LSM14A | ENST00000433627.5 | LSM14A, SCD6 homolog A (S. cerevisiae) |
| 182 | ACACB | ENST00000543201.1 | acetyl-CoA carboxylase beta |
| 183 | FOXP2 | ENST00000408937.3 | forkhead box P2 |
| 184 | RPH3A | ENST00000415485.3 | rabphilin 3A homolog (mouse) |
| 185 | ABCB8 | ENST00000356058.4 | ATP-binding cassette, sub-family B (MDR/TAP), member 8 |
| 186 | SRGAP3 | ENST00000383836.3 | SLIT-ROBO Rho GTPase activating protein 3 |
| 187 | SH3PXD2A | ENST00000369774.4 | SH3 and PX domains 2A |
| 188 | MYPN | ENST00000358913.5 | myopalladin |
| 189 | PLD1 | ENST00000342215.6 | phospholipase D1, phosphatidylcholine-specific |
| 190 | ZFP36L1 | ENST00000555997.1 | ZFP36 ring finger protein-like 1 |
| 191 | ZBTB16 | ENST00000335953.4 | zinc finger and BTB domain containing 16 |
| 192 | IGF1 | ENST00000337514.6 | insulin-like growth factor 1 (somatomedin C) |
| 193 | HSPA12A | ENST00000369209.3 | heat shock 70kDa protein 12A |
| 194 | CD276 | ENST00000318443.5 | CD276 molecule |
| 195 | SLC22A23 | ENST00000436008.2 | solute carrier family 22, member 23 |
| 196 | STXBP1 | ENST00000373302.3 | syntaxin binding protein 1 |
| 197 | TANC2 | ENST00000424789.2 | tetratricopeptide repeat, ankyrin repeat and coiled-coil containing 2 |
| 198 | ATXN7L3 | ENST00000454077.2 | ataxin 7-like 3 |
| 199 | SP6 | ENST00000342234.2 | Sp6 transcription factor |
| 200 | USP15 | ENST00000353364.3 | ubiquitin specific peptidase 15 |
| 201 | KIAA1324 | ENST00000369939.3 | KIAA1324 |
| 202 | SIK2 | ENST00000304987.3 | salt-inducible kinase 2 |
| 203 | FOXP1 | ENST00000318789.4 | forkhead box P1 |
| 204 | MOG | ENST00000376902.3 | myelin oligodendrocyte glycoprotein |
| 205 | SOX6 | ENST00000316399.6 | SRY (sex determining region Y)-box 6 |
| 206 | TCF4 | ENST00000354452.3 | transcription factor 4 |
| 207 | RNASEH2C | ENST00000308418.4 | ribonuclease H2, subunit C |
| 208 | RNF121 | ENST00000393713.3 | ring finger protein 121 |
| 209 | VWA1 | ENST00000338660.5 | von Willebrand factor A domain containing 1 |
| 210 | IP6K1 | ENST00000468463.1 | inositol hexakisphosphate kinase 1 |
| 211 | PAPD5 | ENST00000357464.3 | PAP associated domain containing 5 |
| 212 | DAB2IP | ENST00000408936.3 | DAB2 interacting protein |
| 213 | LAMC1 | ENST00000258341.4 | laminin, gamma 1 (formerly LAMB2) |
| 214 | TGFBRAP1 | ENST00000393359.2 | transforming growth factor, beta receptor associated protein 1 |
| 215 | ESR2 | ENST00000557772.1 | estrogen receptor 2 (ER beta) |
| 216 | ATXN7L3B | ENST00000519948.2 | ataxin 7-like 3B |
| 217 | HELZ | ENST00000358691.5 | helicase with zinc finger |
| 218 | SIRT6 | ENST00000601488.1 | sirtuin 6 |
| 219 | PHF20 | ENST00000439301.1 | PHD finger protein 20 |
| 220 | TSR1 | ENST00000301364.5 | TSR1, 20S rRNA accumulation, homolog (S. cerevisiae) |
| 221 | TYRO3 | ENST00000263798.3 | TYRO3 protein tyrosine kinase |
| 222 | PPP1R16B | ENST00000299824.1 | protein phosphatase 1, regulatory subunit 16B |
| 223 | FAM168A | ENST00000064778.4 | family with sequence similarity 168, member A |
| 224 | NDOR1 | ENST00000427047.2 | NADPH dependent diflavin oxidoreductase 1 |
| 225 | BCL2L2 | ENST00000250405.5 | BCL2-like 2 |
| 226 | C1orf21 | ENST00000235307.6 | chromosome 1 open reading frame 21 |
| 227 | EFNA5 | ENST00000333274.6 | ephrin-A5 |
| 228 | ABHD14B | ENST00000461108.1 | abhydrolase domain containing 14B |
| 229 | ARFGEF2 | ENST00000371917.4 | ADP-ribosylation factor guanine nucleotide-exchange factor 2 (brefeldin A-inhibited) |
| 230 | NFE2L1 | ENST00000585291.1 | nuclear factor, erythroid 2-like 1 |
| 231 | CPLX2 | ENST00000359546.4 | complexin 2 |
| 232 | ANAPC16 | ENST00000299381.4 | anaphase promoting complex subunit 16 |
| 233 | FBXL18 | ENST00000382368.3 | F-box and leucine-rich repeat protein 18 |
| 234 | ABI2 | ENST00000295851.5 | abl-interactor 2 |
| 235 | MACC1 | ENST00000400331.5 | metastasis associated in colon cancer 1 |
| 236 | GGA1 | ENST00000343632.4 | golgi-associated, gamma adaptin ear containing, ARF binding protein 1 |
| 237 | ATG9A | ENST00000396761.2 | autophagy related 9A |
| 238 | AKT3 | ENST00000366539.1 | v-akt murine thymoma viral oncogene homolog 3 |
| 239 | ARHGAP32 | ENST00000524655.1 | Rho GTPase activating protein 32 |
| 240 | SHISA6 | ENST00000441885.3 | shisa family member 6 |
| 241 | SNRPE | ENST00000414487.2 | small nuclear ribonucleoprotein polypeptide E |
| 242 | POM121 | ENST00000395270.1 | POM121 transmembrane nucleoporin |
| 243 | ERC1 | ENST00000355446.5 | ELKS/RAB6-interacting/CAST family member 1 |
| 244 | MORC4 | ENST00000535534.1 | MORC family CW-type zinc finger 4 |
| 245 | C6orf195 | ENST00000296847.3 | chromosome 6 open reading frame 195 |
| 246 | DUOX1 | ENST00000321429.4 | dual oxidase 1 |
| 247 | RPUSD1 | ENST00000565809.1 | RNA pseudouridylate synthase domain containing 1 |
| 248 | GNAI3 | ENST00000369851.4 | guanine nucleotide binding protein (G protein), alpha inhibiting activity polypeptide 3 |
| 249 | SARM1 | ENST00000457710.3 | sterile alpha and TIR motif containing 1 |
| 250 | PLD3 | ENST00000409587.1 | phospholipase D family, member 3 |
| 251 | MAPT | ENST00000344290.5 | microtubule-associated protein tau |
| 252 | TOR2A | ENST00000458505.3 | torsin family 2, member A |
| 253 | KIAA0895L | ENST00000561621.1 | KIAA0895-like |
| 254 | PKNOX1 | ENST00000291547.5 | PBX/knotted 1 homeobox 1 |
| 255 | SAR1A | ENST00000373242.2 | SAR1 homolog A (S. cerevisiae) |
| 256 | ADAMTS18 | ENST00000282849.5 | ADAM metallopeptidase with thrombospondin type 1 motif, 18 |
| 257 | PCDH11X | ENST00000504220.2 | protocadherin 11 X-linked |
| 258 | BRD3 | ENST00000303407.7 | bromodomain containing 3 |
| 259 | MEF2C | ENST00000340208.5 | myocyte enhancer factor 2C |
| 260 | FPGS | ENST00000373245.1 | folylpolyglutamate synthase |
| 261 | JDP2 | ENST00000435893.2 | Jun dimerization protein 2 |
| 262 | TMEM229B | ENST00000557006.1 | transmembrane protein 229B |
| 263 | NFATC1 | ENST00000253506.5 | nuclear factor of activated T-cells, cytoplasmic, calcineurin-dependent 1 |
| 264 | GATS | ENST00000436886.2 | GATS, stromal antigen 3 opposite strand |
| 265 | ADRBK2 | ENST00000324198.6 | adrenergic, beta, receptor kinase 2 |
| 266 | PAX5 | ENST00000358127.4 | paired box 5 |
| 267 | BSN | ENST00000296452.4 | bassoon presynaptic cytomatrix protein |
| 268 | GRIN1 | ENST00000371561.3 | glutamate receptor, ionotropic, N-methyl D-aspartate 1 |
| 269 | BTBD19 | ENST00000453418.1 | BTB (POZ) domain containing 19 |
| 270 | XIRP1 | ENST00000396251.1 | xin actin-binding repeat containing 1 |
| 271 | CREB3L3 | ENST00000602147.1 | cAMP responsive element binding protein 3-like 3 |
| 272 | RNF150 | ENST00000515673.2 | ring finger protein 150 |
| 273 | TTC39A | ENST00000530004.1 | tetratricopeptide repeat domain 39A |
| 274 | MARK4 | ENST00000300843.4 | MAP/microtubule affinity-regulating kinase 4 |
| 275 | LRCH4 | ENST00000310300.6 | leucine-rich repeats and calponin homology (CH) domain containing 4 |
| 276 | ZNF652 | ENST00000362063.2 | zinc finger protein 652 |
| 277 | HAUS3 | ENST00000243706.4 | HAUS augmin-like complex, subunit 3 |
| 278 | WNK1 | ENST00000315939.6 | WNK lysine deficient protein kinase 1 |
| 279 | CADM1 | ENST00000452722.3 | cell adhesion molecule 1 |
| 280 | ZBTB7B | ENST00000368426.3 | zinc finger and BTB domain containing 7B |
| 281 | DVL3 | ENST00000313143.3 | dishevelled segment polarity protein 3 |
| 282 | UBE2K | ENST00000261427.5 | ubiquitin-conjugating enzyme E2K |
| 283 | YIF1B | ENST00000337679.8 | Yip1 interacting factor homolog B (S. cerevisiae) |
